# Transplant Trial Watch

**DOI:** 10.3389/ti.2024.13360

**Published:** 2024-06-24

**Authors:** Simon R. Knight, John M. O’Callaghan, John Fallon

**Affiliations:** ^1^ Centre for Evidence in Transplantation, Nuffield Department of Surgical Sciences, University of Oxford, Oxford, United Kingdom; ^2^ Oxford Transplant Centre, Churchill Hospital, Oxford, United Kingdom; ^3^ University Hospitals Coventry & Warwickshire, Coventry, United Kingdom

**Keywords:** randomised controlled trial, lung transplantation, kidney transplantation, extracorporeal membrane oxygenation (ECMO), mycophenolate mofetil

To keep the transplantation community informed about recently published level 1 evidence in organ transplantation ESOT and the Centre for Evidence in Transplantation have developed the Transplant Trial Watch. The Transplant Trial Watch is a monthly overview of 10 new randomised controlled trials (RCTs) and systematic reviews. This page of Transplant International offers commentaries on methodological issues and clinical implications on two articles of particular interest from the CET Transplant Trial Watch monthly selection. For all high quality evidence in solid organ transplantation, visit the Transplant Library: www.transplantlibrary.com

Randomised Controlled Trial 1 Randomized Trial of Routine Versus On-Demand Intraoperative Extracorporeal Membrane Oxygenation in Lung Transplantation: a Feasibility Study.

*by Nasir, B., et al. Journal of Heart and Lung Transplantation 2024 [record in progress].*

## Aims

Assess the feasibility of undertaking a multicentre RCT to compare two strategies of intraoperative mechanical circulatory support (routine ECMO versus on-demand ECMO) during lung transplantation.

## Interventions

Standard of care being routine ECMO versus the intervention of on-demand ECMO utilised when required during transplantation.

## Participants

28 adult, lung only, primary transplant recipients where cardiopulmonary support was not mandatory were randomised.

## Outcomes

The outcome measures were death, primary graft dysfunction (PGD), bleeding, cannulation site complications, and hypoperfusion-related complications (e.g., AKI, stroke, mesenteric ischemia).

## Follow-Up

30 days

## CET Conclusion

*by John Fallon*

This is a small, randomised feasibility study conducted in a single Canadian lung transplant centre with the aim of designing a large multicentre RCT to definitively assess the use of routine versus on-demand ECMO during lung only transplantation. They perform a sensible power calculation based on the Blackwelder method and discussion across all Canadian lung centres with regards historic data and possible effect sizes, giving a needed trial size of 310 patients in each arm. They apply this to collected data on local and national transplant numbers to assess are reasonable study period and recruitment window. Based on their contribution to national transplant number they go on to generate an aim within their centre during a 6-month feasibility recruitment period. They determine their trial would likely be feasible and at low risk of failure if they randomised 19 participants with fewer than 5% loss-to-follow up and less than 10% protocol violations within the 6 months. During the feasibility study period they successfully randomise 28 patients. While the numbers are insufficient to comment on the two interventions, they demonstrate that over the proposed 3-year study period with all 4 Canadian lung transplant centres it is highly likely the trial could be achieved, and a definitive answer found. This is a commendable feasibility study, with complex interventions with potentially small effect sizes, it is crucial that should one embark on the cost, effort, and patient recruitment for such trials that the risk of failure is minimised as far as possible. Strategies such as a well-thought-out simple feasibility studies are key to larger trial successes.

## Jadad Score

3.

## Data Analysis

Strict intention-to-treat analysis.

## Allocation Concealment

Yes.

## Trial Registration

ClinicalTrials.gov—NCT05505422.

## Funding Source

No funding received.

Randomised Controlled Trial 2 Decreased Mycophenolate Mofetil Hampers Antibody Responses to a Broad Range of Vaccinations in Kidney Transplant Recipients: Results From a Randomized Controlled Study.

*by Fatly, Z. A., et al. Journal of Infection 2024; 88(3): 106133.*

## Aims

This study aimed to investigate whether discontinuing mycophenolate mofetil (MMF) 3 months prior to vaccination would improve vaccination responses in renal transplant recipients using tacrolimus.

## Interventions

Participants were randomised to either tacrolimus monotherapy (TACmono) or to tacrolimus with MMF (TAC/MMF).

## Participants

79 kidney transplant recipients.

## Outcomes

The main outcomes of interest were responses to pneumococcal, tetanus and influenza vaccination; relation between pneumococcal, tetanus, and influenza vaccination responses; clinical differences in vaccination responders versus non-responders; correlation between SARS-CoV-2, pneumococcal, and tetanus vaccination responses; and effect of Co-administering of influenza vaccines on pneumococcal and tetanus serological vaccination responses.

## Follow-Up

21 days post-vaccination.

## CET Conclusion

*by John O’Callaghan*

This is a very interesting paper following on from a randomised controlled trial that has been previously published. In the initial trial kidney transplant recipients were randomised to continue on tacrolimus monotherapy instead of a tacrolimus and mycophenolate combination (de Weerd et al. Transpl Int. 2022 October 24; 35:10839). In the present paper these two cohorts were monitored for their serological responses to key vaccinations: pneumococcus, tetanus, influenza. The results show a very significant difference in the vaccine responses when assessing each vaccine individually, with tacrolimus monotherapy being beneficial. In addition, only 7% responded adequately to all of pneumococcus, tetanus and influenza vaccines whilst on tacrolimus and mycophenolate monotherapy. In this group 40% responded inadequately to all 3 of these vaccinations. In contrast, 100% of those on tacrolimus monotherapy responded to at least one of the vaccines. No significant differences were seen in the clinical outcome of responders versus non-responders, but at this level of analysis the study becomes too small for the outcomes being assessed (patient survival, infection-related death and antibiotic use. In addition a small number of those in the study received the sars-cov2 vaccine when it became available. Sars-cov2 antibody levels were significantly lower following vaccination in the tacrolimus and mycophenolate group compared to the tacrolimus monotherapy group. The inhibition of both B and T-cell responses by mycophenolate hampers the body’s response to vaccination and the effect is clearly shown by this study. However, in this study, the response was only moderately dose dependent, so reducing mycophenolate dosing does not help significantly with vaccine responses, compared to stopping the drug 3 months prior to vaccinations. If reducing immune suppression is not possible then this study highlights the importance of vaccination prior to transplantation.

## Trial Registration

EudraCT nr.: 2014-001372-66.

## Funding Source

Non-industry funded.

## Clinical Impact Summary

*by Simon Knight*

The COVID-19 pandemic served as a reminder not just of the susceptibility of immunosuppressed patients to infection, but also to their reduced ability to show serological response to vaccination or infection. The large, UK-based Melody study included nearly 10,000 solid organ transplant recipients, and demonstrated that patients on steroid or mycophenolate mofetil (MMF) therapy were far less likely to develop an antibody response to SARS-CoV-2 [1]. Those on triple immunosuppression (antiproliferative, calcineurin inhibitor (CNI) and steroids) were significantly less likely to respond than those receiving dual or monotherapy, suggesting that it is overall immunosuppression burden that is important, rather than specific agents.

The potential benefits of immunosuppression minimisation have been well studied, largely focussing on either the metabolic benefits of steroid withdrawal, or the reduction in renal injury, infection and malignancy risk with CNI minimisation [2, 3]. Immunosuppression minimisation may have the additional benefit of improving vaccination responses in vulnerable patients.

In a recent pilot study, researchers from Erasmus Medical Centre in the Netherlands investigated the ability to withdraw MMF in low immunological-risk recipients by 9 months following renal transplant [4]. A pre-planned sub-study investigated responses to the pneumococcal, tetanus and influenza vaccines at 12-month post-transplant [5]. Serological vaccination response was measured for all three vaccinations. Adequate serological responses were seen in 74%, 82%, and 71% of tacrolimus monotherapy patients for the pneumococcal, tetanus and influenza vaccines respectively, in comparison to 43%, 35%, and 20% patients remaining on dual therapy with MMF.

These results suggest that the ability to respond to vaccination is significantly improved within 3-months of MMF withdrawal, an effect that spans different vaccine types. It highlights the importance of vaccination prior to transplant where possible and provides more ammunition for consideration of immunosuppression minimisation in lower-risk transplant recipients. The study is too small to demonstrate whether improved vaccine response translates to measurable clinical benefit, but nonetheless provides further evidence of the importance of immunosuppressive load on vaccine responses.

### Clinical Impact

3/5.

## References

[1] Pearce FA Lim SH Bythell M Lanyon P Hogg R Taylor A Antibody Prevalence after Three or More COVID-19 Vaccine Doses in Individuals Who Are Immunosuppressed in the UK: A Cross-Sectional Study from MELODY. Lancet Rheumatol (2023) 5:e461–e473. 10.1016/S2665-9913(23)00160-1 38251578

[2] Pascual J Royuela A Galeano C Crespo M Zamora J. Very Early Steroid Withdrawal or Complete Avoidance for Kidney Transplant Recipients: A Systematic Review. Nephrol Dial Transplant (2012) 27:825–32. 10.1093/ndt/gfr374 21785040

[3] Karpe KM Talaulikar GS Walters GD. Calcineurin Inhibitor Withdrawal or Tapering for Kidney Transplant Recipients. Cochrane Database Syst Rev (2017) 7:CD006750. 10.1002/14651858.CD006750.pub2 28730648 PMC6483545

[4] de Weerd AE Fatly ZA Boer-Verschragen M Kal-van Gestel JA Roelen DL Dieterich M Tacrolimus Monotherapy Is Safe in Immunologically Low-Risk Kidney Transplant Recipients: A Randomized-Controlled Pilot Study. Transpl Int (2022) 35:10839. 10.3389/ti.2022.10839 36353052 PMC9637544

[5] Fatly ZA Betjes MGH Dik WA Fouchier RAM Reinders MEJ de Weerd AE. Mycophenolate Mofetil Hampers Antibody Responses to a Broad Range of Vaccinations in Kidney Transplant Recipients: Results from a Randomized Controlled Study. J Infect (2024) 88:106133. 10.1016/j.jinf.2024.106133 38432583

